# Plasma Exosomal S1PR5 and CARNS1 as Potential Non-invasive Screening Biomarkers of Coronary Heart Disease

**DOI:** 10.3389/fcvm.2022.845673

**Published:** 2022-06-28

**Authors:** Feng Xiong, Rui Mao, Ruohan Zhao, Lijuan Zhang, Kunyue Tan, Chunxia Liu, Shuzhen Wang, Min Xu, Yi Li, Tongtong Zhang

**Affiliations:** ^1^Department of Cardiology, Cardiovascular Institute of Chengdu, The Third People's Hospital of Chengdu, Chengdu, China; ^2^Department of Dermatology, Xiangya Hospital, Central South University, Changsha, China; ^3^The Center of Gastrointestinal and Minimally Invasive Surgery, The Third People's Hospital of Chengdu, Chengdu, China; ^4^Department of Radiology, The Third People's Hospital of Chengdu, Chengdu, China; ^5^Medical Research Center, The Third People's Hospital of Chengdu, Chengdu, China; ^6^Department of General Surgery, Center of Gastrointestinal and Minimally Invasive Surgery, The Third People's Hospital of Chengdu, Affiliated Hospital of Southwest Jiaotong University, Chengdu, China

**Keywords:** coronary heart disease, mRNA, sEVs, lipid metabolism, genomics

## Abstract

**Background:**

Early diagnosis and treatment significantly improve the prognosis of coronary heart disease (CHD), but no convenient screening tools are available. This study aims to find potential non-invasive screening biomarkers of coronary heart disease.

**Method:**

We performed microarray analysis to investigate the mRNA expression levels in Small extracellular vesicles (sEVs) and screen significantly differentially expressed mRNAs in CHD patients vs. non-CHD patients. We then performed quantitative real-time polymerase chain reaction (qRT-PCR) to validate the microarray results, and we calculated the correlations between expression levels and clinicopathological data. Microarray analysis identified 72 downregulated mRNAs and 31 upregulated mRNAs in CHD patients relative to non-CHD patients.

**Results:**

From the study, we found that upregulated sphingosine-1-phosphate receptor 5 (S1PR5) and downregulated carnosine synthase 1 (CARNS1) had the most significant differences between the patient group and the control group. S1PR5 expression was correlated with diabetes, heart rate, triglycerides, total cholesterol, low-density lipoprotein cholesterol, apolipoprotein B, and fasting blood glucose (*P* < 0.05). CARNS1 level was correlated with uric acid (UA) (*P* < 0.05). Overexpressed S1PR5 and downregulated CARNS1 were independent risk factors for CHD. The area under the receiver operating characteristic curve (AUC) of S1PR5 was 0.838 for diagnosing CHD; the AUC of CARNS1 was 0.883 for non-CHD; and the AUC of S1PR5 plus CARNS1 was 0.921 for CHD.

**Conclusions:**

Microarray analysis showed that upregulated S1PR5 and downregulated CARNS1 in sEVs have the potential to become non-invasive biomarkers for CHD screening.

## Introduction

Coronary heart disease (CHD) is the most common cardiovascular disease in the world and is the cause of 50% of heart failure (1), with the highest mortality and morbidity among cardiovascular diseases. It is associated with a high public health burden (2, 3). Early diagnosis and treatment of CHD help delay the development of heart failure and significantly improve the prognosis. Coronary angiography is the gold standard for the diagnosis of CHD, but it is an invasive procedure that may cause vessel injury and intraoperative infection. Coronary CT angiography (CTA) shows the stenosis of coronary arteries, but it entails radiation exposure and a risk of contrast allergy. These two common diagnostic methods are not suitable for CHD screening, while myocardial markers and electrocardiogram (ECG), commonly used during routine check-up, are susceptible to non-cardiogenic factors. At present, no convenient screening tools are available for CHD.

Small extracellular vesicles (sEVs) are 30–150-nm-diameter phospholipid-bilayered vesicles produced in the cells. They transmit information *via* paracrine signaling and carry various biologically active substances, including RNAs, such as messenger RNAs (mRNAs) and long non-coding RNAs (lncRNAs) (4). Their RNA content is relatively stable due to absence of nucleases and thus may be used as screening biomarkers and treatment targets of cancer and cardiovascular diseases (5, 6). mRNAs transmit information from the nucleus to the cytoplasm to encode proteins with cellular function. lncRNAs regulate protein synthesis by regulating mRNAs (7). lncRNAs are highly stable molecules with a long half-life, making them ideal markers for disease screening and a preferred treatment target (8, 9). In sEVs, however, mRNAs are more stable than in the cell, and the mRNA level reflects their regulation by lncRNAs, giving them a more direct correlation with coded protein levels and cellular functions. mRNAs can be used for diagnostic and prognostic assessment of colorectal cancer (9). We speculate that mRNAs in sEVs have a similar potential value for the diagnosis of CHD.

## Results

### Identification of sEVs in CHD Patients

**Figure 1:**
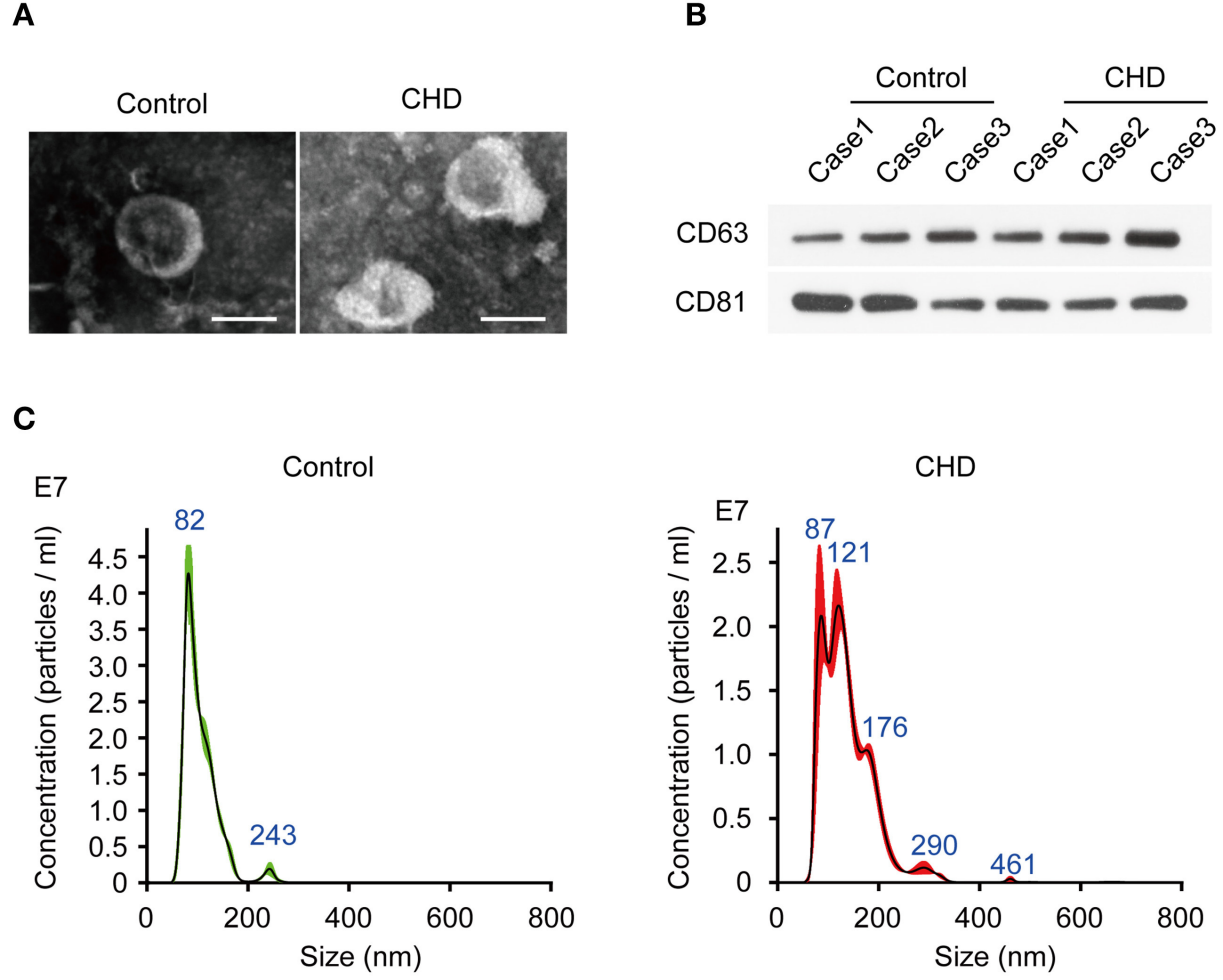
Identification of plasma exosomes in CHD patients. **(A)** The representative images of plasma exosomes derived from CHD group and the control group patients analyzed by transmission electron microscopy. Scale bar, 100 nm. **(B)** The positive markers of exosomes, CD63 and CD81 were detected in of plasma exosomes by Western blot. **(C)** Nanosight particle tracking analysis (NTA) of plasma exosomes derived from CHD group and the control group patients.

First, we extracted monocytes from plasma, and the result of analysis of monocytes can refer to Supplementary Figure S1 of our published article (10). Through transmission electron microscopy (TEM), we found that the sEVs were ~100 nm in diameter and conformed to the known shape of sEVs. Next, we detected the sizes of sEVs by the NTA method and found that sEVs from monocytes were between 40 and 150 nm in diameter (Figure 1A), which was consistent with common sizes of known sEVs and with the TEM results (Figure 1C). Western blot showed that the protein markers of sEVs CD63 and CD81 were more highly expressed in sEVs than in monocytes (Figure 1B). Thus, we successfully extracted sEVs secreted by monocytes isolated from coronary atherosclerosis and control patients.

### Different mRNA Expression Patterns in sEVs From CHD and Control Individuals

**Figure 2:**
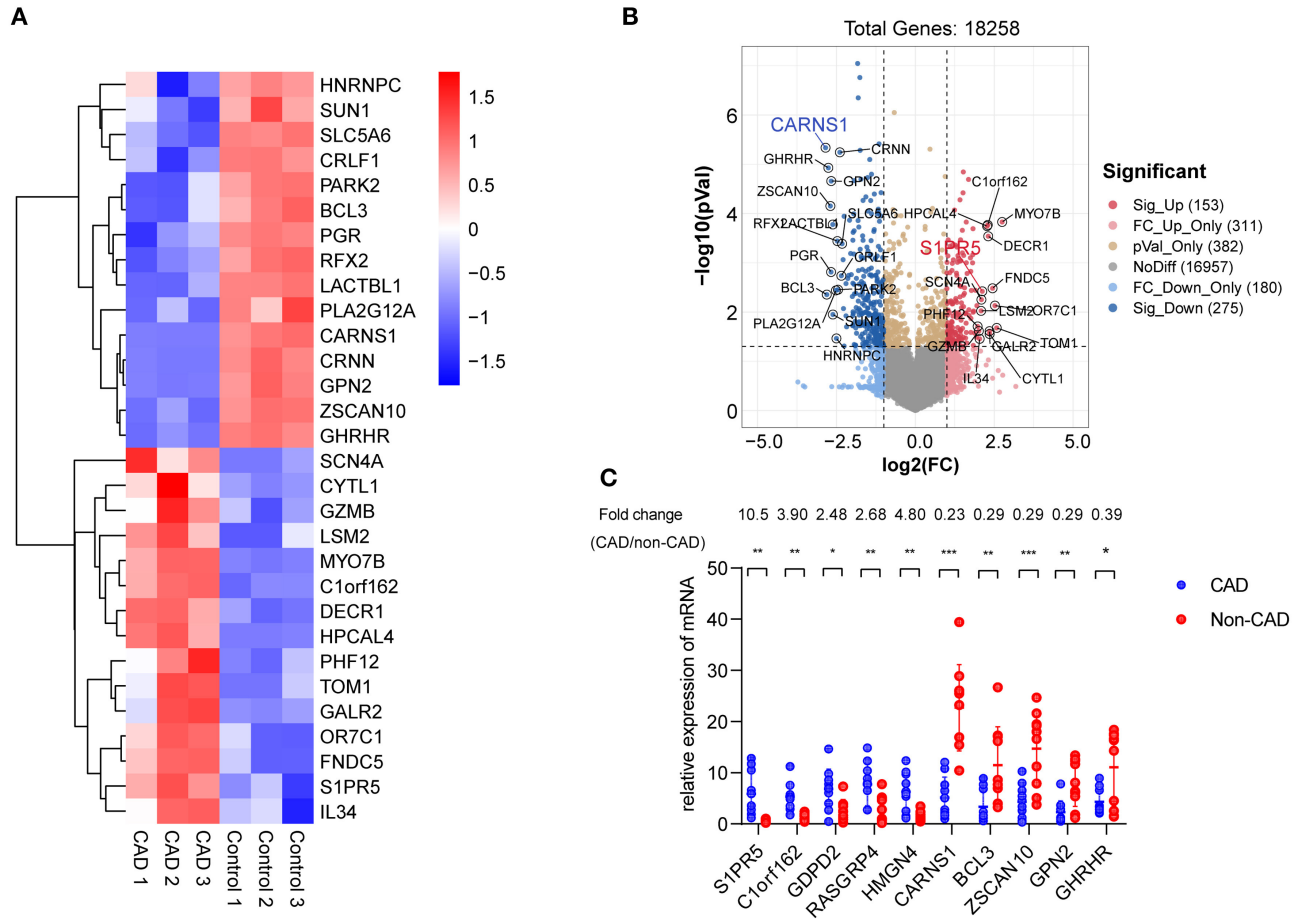
Identification of differentially expressed mRNAs in plasma exosomes derived from CHD patients. **(A)** Heat map showing hierarchical clustering analysis of mRNAs detected in CHD group and the control group patients. **(B)** Volcano map showing the distribution of differential mRNAs according to their *p*-values and fold-changes. Candidates with *p* < 0.05 and |log 2(fold-change) |≥1 are considered differential. The molecules with the most significant differences are marked on the volcano map. **(C)** Differential expression of 10 mRNAs was validated in plasma exosomes derived from CHD group and the control group patients using qRT-PCR. Data are presented as means ± SD; significant difference was identified with Student's *t*-test. *P < 0.05; **P < 0.01 and ***P < 0.005; ns, not significant.

**Figure 3:**
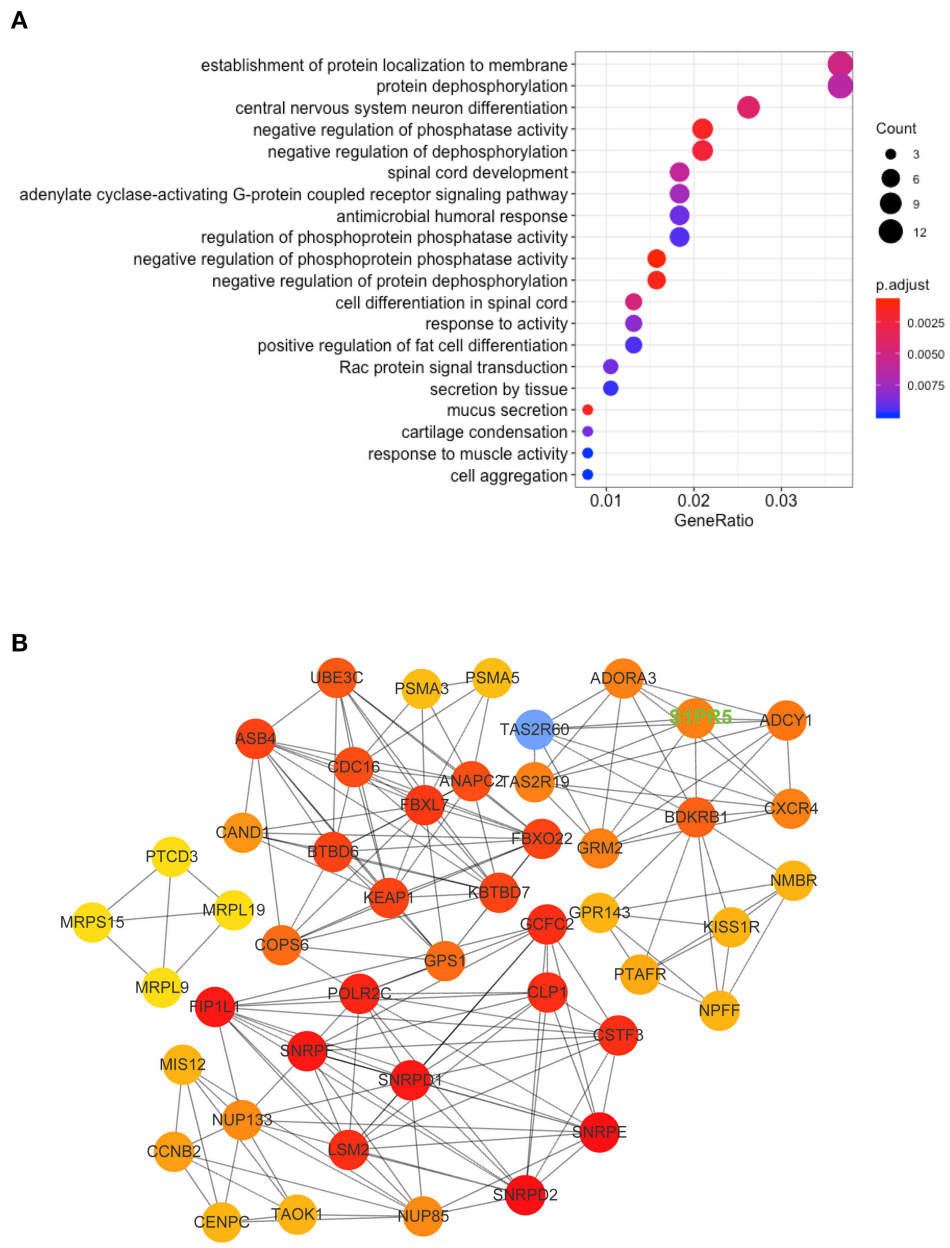
KEGG Pathway and mRNA interaction network analysis based on the validated mRNA candidates. **(A)** KEGG pathway enrichment analysis of these mRNAs with the 20 highest enrichment scores. **(B)** mRNA interaction network based on the validated mRNA candidates. Every circle represents a mRNA. The darkness of the color denotes the strength of interaction. The key molecule we studied, S1PR5, has been highlighted in uppercase green fonts on the picture.

Table 1 shows the general clinical data of the control group and the patient group. Microarray analysis identified differentially expressed mRNAs between the control group and the patient group, including 72 downregulated mRNAs and 31 upregulated mRNAs in the patient group. See the heat map in Figure 2A and the volcano plot in Figure 2B for the gene expression profiles. We verified the first five molecules with the most significant upregulation and downregulation by QRT-PCR. The preliminary validation showed that the most differentially expressed mRNAs were the upregulated S1PR5 and the downregulated CARNS1 in the patient group (Figure 2C). Kyoto Encyclopedia of Genes and Genomes (KEGG) analysis was performed to investigate the pathological pathways of the significantly differentially expressed mRNAs (Figure 3A). The results showed that these mRNAs were related to localization of membrane proteins, protein phosphorylation and dephosphorylation, G-coupled receptor pathways responsible for activating adenylate cyclase, and adipocyte differentiation. Figure 3B shows the interactive network of isolated mRNAs inside the sEVs.

**Table 1:** Information of CHD patients and non-CHD patients.

**Parameter**	**CAD (*n* = 120)**	**Non-CAD (*n* = 121)**	**Z/t/χ^2^**	***p***
	**P50 (P25**~**P75)/** $\bar{\text{x}}\pm$ **t**	**P50 (P25**~**P75)/** $\bar{\text{x}}\pm$ **t**		
Age (years)	57 (51.1~62)	56 (53.2~63)	−0.459	0.646
BMI (Kg/m^2^)	24.47 (22.07~26.75)	25.16 (23.05~26.36)	−1.095	0.274
Smoking	Current 58 (48.3%)	44 (30.9%)	10.32	<0.01^**^
Alcohol	Current 27 (22.5%)	23 (20.9%)	3.13	0.21
HBP	85 (70.8%)	69 (57.0%)	4.98	0.03^*^
DM	38 (31.7%)	26 (21.7%)	3.07	0.08
SBP (mmHg)	124 (115~136.75)	123 (113.75~134)	−0.878	0.380
DBP (mmHg)	76.01 ± 9.98	77.22 ± 10.00	−0.93	0.36
HR (bpm)	75 (68~80)	72.5 (67~80.25)	−0.382	0.703
LVD (mm)	46 (42~50)	46 (42.5~48)	−0.259	0.796
EF (%)	60.5 (55~63)	61 (57~64)	−1.396	0.163
cTNT (pg/ml)	10.06 (7.57~21.26)	8.09 (5.47~14.2)	−2.376	0.018^*^
CKMB (U/L)	10.7 (8.75~13.63)	10.6 (7.85~13.8)	−1.055	0.291
BNP (pg/ml)	58.3 (24.1~115.13)	41.45 (22~98.75)	−0.645	0.519
HCY (umol/l)	12.3 (10.6~14.9)	11.45 (9.2~14.7)	−2.192	0.028^*^
LPA (mg/l)	115.5 (60.53~369.65)	79 (43.4~204.8)	−2.762	0.006^**^
BUN (mmol/l)	5.73 (4.65~6.87)	5.275 (4.63~6.445)	−1.325	0.185
Scr (umol/l)	76.05 (68.78~85.6)	73.9 (61.98~83.35)	−1.716	0.086
UA (umol/l)	366 (315.3~441.48)	354 (291.55~416.35)	−1.381	0.167
β2-Gm (mg/l)	1.97 (1.66~2.68)	1.89 (1.68~2.12)	−1.685	0.092
Fglu (mmol/l)	5.34 (4.77~6.6)	5.54 (5.17~6.27)	−1.376	0.169
TG (mmol/l)	1.29 (1~2.09)	1.32 (1.02~1.74)	−0.087	0.931
TC (mmol/l)	4.01 (3.33~5.09)	4.29 (3.64~4.89)	−1.142	0.254
HDL-c (mmol/l)	1.18 (0.99~1.3)	1.22 (1.09~1.47)	−2.155	0.031^*^
LDL-c (mmol/l)	2.38 (1.83~3.13)	2.59 (2.14~3.16)	−1.569	0.117
Apo a (g/l)	1.29 (1.12~1.49)	1.31 (1.13~1.62)	−1.036	0.300
Apo b (g/l)	0.71 (0.58~0.98)	0.75 (0.61~0.93)	−0.511	0.610
Hba1c (%)	6.6 (6.1~7.7)	5.9 (5.6~6.2)	−3.891	<0.001^***^

*PS:*

*** *P < 0.005*,

** *P < 0.01*,

* *P < 0.05*.

### Diagnostic Value of S1PR5 and CARNS1

**Figure 4:**
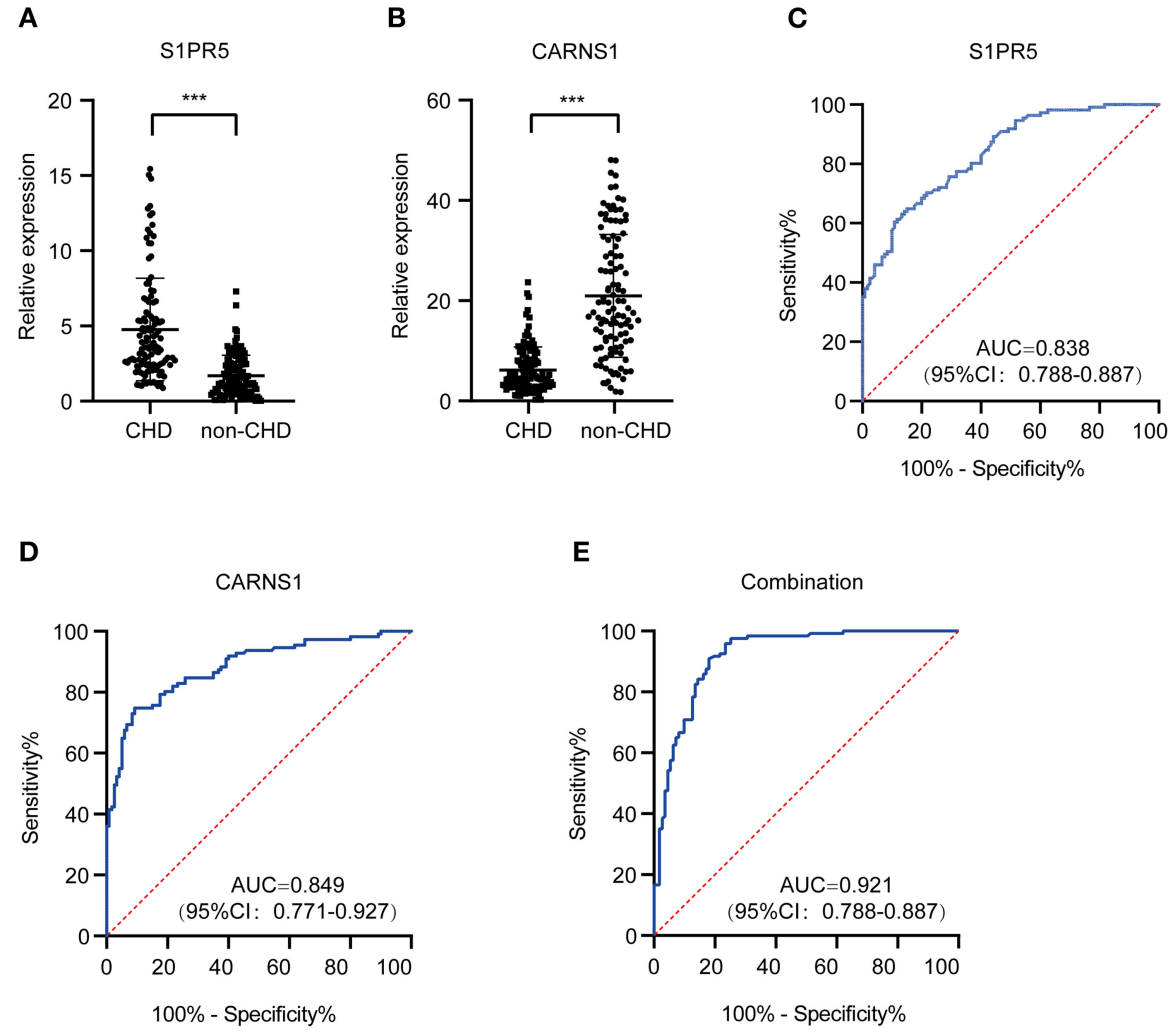
Diagnostic value of plasma exosomal *S1PR5* and *CARNS1* in CHD patients. qRT-PCR analysis of expression of S1PR5 **(A)** and CARNS1 **(B)** in a large sample of CHD patients (*n* = 120) and healthy controls (*n* = 121). ROC curve analysis of S1PR5 **(C)**, CARNS1 **(D)** and combination of S1PR5 and CARNS1 **(E)** for discrimination of CHD patients (*n* = 120) from healthy controls (*n* = 121). ***P < 0.005.

qRT-PCR validation in more subjects showed significantly differential expression of S1PR5 and CARNS1 between the patient group and the control group (*P* < 0.05) (Figures 4A,B). ROC analysis showed that the area under the curve (AUC) of S1PR5 was 0.838 [95% confidence interval (CI): 0.788–0.887, *P* < 0.05] for diagnosing CHD (Figure 4C). When S1PR5 expression was >1.945, it had a sensitivity of 0.850 and specificity of 0.649 for diagnosing CHD. CARNS1 expression was negatively correlated with CHD. Its AUC was 0.849 (95% CI: 0.771–0.927, *P* < 0.05) for identifying non-CHD (Figure 4D). When CARNS1 was > 0.649, it had a sensitivity of 0.748 and specificity of 0.908 for non-CHD. S1PR5 plus CARNS1 had an AUC of 0.921 (Figure 4E).

### Abnormal Expression of S1PR5 or CARNS1 Predict Aggressive Clinical-Pathological Characteristics in CHD Patients

To investigate whether S1PR5 and CARNS1 are associated with CHD, spearman analysis was performed to analyze the correlations between S1PR5 and CARNS1 and CHD-related factors (Table 2). In the patient group, S1PR5 was correlated with diabetes, heart rate, triglycerides (TG), total cholesterol (TC), low-density lipoprotein cholesterol (LDL-C), apolipoprotein B (ApoB), and fasting blood glucose (*P* < 0.05); CARNS1 was correlated with uric acid (UA) (*P* < 0.05). Diabetes, TG, TC, LDL-C, ApoB, and UA are CHD-related metabolism indicators, suggesting S1PR5 and CARNS1 may be related to the pathogenesis of CHD.

**Table 2:** Correlation between baseline characteristic and mRNAs level in CHD patients.

**Parameter**	**S1PR5**		**CARNS1**	
	**Coefficient**	***P***	**Coefficient**	***P***
Age (years)	−0.03	0.75	−0.01	0.95
HBP	−0.084	0.362	−0.096	0.297
DM	0.332	<0.001^***^	−0.155	0.091
BMI (kg/m^2^)	−0.23	0.14	−0.01	0.93
HR (bpm)	0.25	<0.01^**^	−0.05	0.60
SBP (mmHg)	−0.06	0.51	−0.14	0.13
DBP (mmHg)	−0.02	0.81	0.03	0.72
Myo (ng/ml)	−0.24	0.07	0.20	0.13
cTnT (pg/ml)	0.06	0.52	0.07	0.51
CKMB (U/L)	0.01	0.94	0.02	0.83
BNP (pg/ml)	−0.04	0.71	0.14	0.18
TG (mmol/L)	0.29	0.002^***^	−0.09	0.34
TC (mmol/L)	0.22	0.02^*^	−0.06	0.53
HDL-C (mmol/L)	−0.02	0.85	0.13	0.18
LDL-C (mmol/L)	0.20	0.04^*^	−0.10	0.32
Apo a (g/L)	0.06	0.56	0.11	0.25
Apo b (g/L)	0.22	0.02^*^	−0.06	0.57
LP a (mg/L)	−0.14	0.15	−0.04	0.65
Fglu (mmol/L)	0.20	0.03^*^	−0.04	0.70
HbA1c (%)	0.08	0.70	−0.25	0.18
HCY (ummol/L)	−0.04	0.71	−0.03	0.79
Scr (umol/L)	−0.08	0.38	0.04	0.69
UA (ummol/L)	−0.05	0.56	0.20	0.03^*^
β2-GM (mg/L)	0.10	0.30	−0.03	0.72
Syntex	0.05	0.58	0.14	0.14
LVD (mm)	−0.07	0.47	−0.02	0.88
EF (%)	0.06	0.56	−0.02	0.87

*PS:*

*** *P < 0.005*,

** *P < 0.01*,

* *P < 0.05*.

Logistic regression analysis showed that S1PR5 and CARNS1 were independent factors for CHD (Table 3). After adjusting for age, body mass index, sex, hypertension, diabetes, smoking, TC, LDL-C, ApoA, and ApoB, S1PR5 was correlated with CHD (odds ratio 2.164, 95% CI: 1.670–2.804, *P* < 0.05), suggesting that higher S1PR5 expression may increase the risk of CHD. After adjusting for the above factors, CARNS1 was correlated with CHD (odds ratio 0.788, 95% CI: 0.730–0.851, *P* < 0.05), suggesting that CARNS1 may be a protective factor against CHD.

**Table 3:** Univariate and multivariate logistic regression analysis to identify mRNAs as independent predictors of CHD.

**Variable**	**S1PR5**				**CARNS1**			
	**OR**	**95%CI**		***P***	**OR**	**95%CI**		***P***
		**Lower**	**Upper**			**Lower**	**Upper**	
Univariate	2.165	1.721	2.722	<0.001	0.795	0.747	0.846	<0.001^***^
analysis							
Model 1	2.192	1.726	2.783	<0.001	0.802	0.752	0.855	<0.001^***^
Model 2	2.224	1.747	2.831	<0.001	0.802	0.752	0.856	<0.001^***^
Model 3	2.233	1.753	2.846	<0.001	0.805	0.755	0.858	<0.001^***^
Model 4	2.183	1.715	2.779	<0.001	0.808	0.758	0.862	<0.001^***^
Model 5	2.250	1.749	2.894	<0.001	0.804	0.751	0.860	<0.001^***^
Model 6	2.169	1.682	2.799	<0.001	0.809	0.755	0.866	<0.001^***^
Model 7	2.176	1.678	2.823	<0.001	0.787	0.729	0.850	<0.001^***^
Model 8	2.164	1.670	2.804	<0.001	0.788	0.730	0.851	<0.001^***^

*PS:*

*** *P < 0.005*.

*Model 1 include age, BMI, gender.*

*Model 2 include age, BMI, gender, HBP*.

*Model 3 include age, BMI, gender, HBP, DM*.

*Model 4 include age, BMI, gender, HBP, DM, smoking*.

*Model 5 include age, BMI, gender, HBP, DM, smoking, Apo b*.

*Model 6 include age, BMI, gender, HBP, DM, smoking, Apo b, LDL-c*.

*Model 7 include age, BMI, gender, HBP, DM, smoking, Apo b, LDL-c, TC*.

*Model 8 include age, BMI, gender, HBP, DM, smoking, Apo b, LDL-c, TC, Apo a*.

## Discussion

This study identified differentially expressed mRNAs in peripheral sEVs between CHD patients and non-CHD patients. S1PR5 (upregulated) and CARNS1 (downregulated) were the most significantly differentially expressed in CHD patients. Spearman correlation analysis between their expression and clinical parameters showed that S1PR5 was associated with diabetes, heart rate, TG, TC, LDL-C, ApoB, and fasting blood glucose, while CARNS1 was associated with uric acid, suggesting S1PR5 and CARNS1 may be related to CHD. Binary logistic regression analysis showed that upregulated S1PR5 and downregulated CARNS1 were independent risk factors for CHD.

CHD is related to many factors, and early diagnosis and intervention are critical for improving the prognosis. S1PR5 and CARNS1 may be used as biomarkers for CHD screening and are superior to ECG (11) and the exercise ECG stress test (12), two of which currently used for CHD screening. For S1PR5 and CARNS1, the sensitivities for detecting CHD were 0.29 and 0.79, and the specificities were 0.79 and 0.80, which were slightly inferior to the values of coronary CTA (sensitivity: 0.92, specificity: 0.75) (13). However, S1PR5 and CARNS1 tests are easy to perform, with no radiation exposure or risk of contrast allergy. Moreover, S1PR5 and CARNS1 has better diagnostic value than other selected CHD biomarkers, such as has-circ-0124644, has-circ-0001879, has-circ-0004104, hsa-miR-584-5p, miR-1, miR-133, and TCONS-00029157 5 (14–17).

This study showed that upregulated S1PR5 mRNA in sEVs was an independent risk factor for CHD. As a member of the S1P/S1PR signaling pathway, S1PR5 is mainly expressed in the nervous system, skin, and natural killer cells (18). As is shown in the KEGG pathway, S1PR5 encodes one of the G-protein coupled receptor which localize on the membrane. No clinical studies have investigated the correlation between S1PR5 and cardiovascular disease (19, 20). As a regulator of fibrosis, S1P activates the S1PR receptor on myocardial cells to regulate fibrosis *via* different downstream signaling pathways, such as the S1P/S1PR1-cAMP pathway (21). Once activated, the S1P/S1PR pathway increases the permeability of vascular endothelial cells and the expression of ICAM-1/VCAM-1, thereby mediating cellular inflammatory responses (22, 23). In alveolar macrophages, S1PR5 overexpression may decrease efferocytosis of macrophages, resulting in accumulation of dead cells and chronic inflammatory diseases (24). This effect may also be present in cardiovascular diseases (25). These data indicate that S1PR5 may promote atherosclerosis by regulating myocardial fibrosis pathways, endothelial cell permeability, and macrophage efferocytosis.

The expression of carnosine synthase–encoding CARNS1 was downregulated in CHD patients, and CARNS1 overexpression was a protective factor against CHD. CARNS1-encoded carnosine plays an important role in the regulation of plasma lipid metabolism in obese mice and obese adults (26–29). Caruso et al. showed that in atherosclerosis, carnosine regulates the energy state of macrophages during oxidative stress and inflammatory responses, restores and/or increases the expression of antioxidant enzymes (Gpx1, SOD-2, Cat), enhances macrophage-mediated degradation of LDL-C and esterification of cholesterol, and reduces atherosclerosis (30, 31). Moreover, with chelation of metal ions, carnosine upregulates the pro-angiogenic HIF-1α/VEGF pathway and improves blood flow and limb function of the affected limb in mice with lower limb ischemia (32). In short, CARNS1 and its product carnosine may play roles in diseases characterized by elevated oxidative stress and inflammatory response, such as atherosclerosis.

This study has some limitations. First, the sample size was small, so further research is needed to validate the diagnostic efficacy of S1PR5 and CARNS1 mRNAs for diagnosing CHD. Second, the significance of S1PR5 and CARNS1 mRNAs in the prognosis of CHD is not clear, and more follow-up data are needed to validate their roles. In summary, this study shows that S1PR5 may be a genetic marker for the diagnosis of CHD. Its measurement is non-invasive and easy to perform and can be used for preliminary CHD screening to promote early intervention and guidance for outpatient follow-up of CHD patients. CARNS1 helps identify non-CHD patients. The combination of S1PR5 and CARNS1 improves the diagnostic efficacy for CHD.

## Materials and Methods

### Patient Screening

The ethics committee of The Third People's Hospital of Chengdu approved to carry out the study within its facilities [Ethical Application Ref: The Third People's Hospital of Chengdu ethics (2019) S−93]. The written form of consent was acquired. A total of 120 patients admitted to the Department of Cardiology, and diagnosed with CHD by coronary angiography between June 2019 and January 2020 were included in the patient group. The diagnoses included chronic CHD, stable angina pectoris, and acute coronary syndrome. Coronary angiography showed ≥50% stenosis of the left coronary artery trunk or ≥75% stenosis of subepicardial coronary arteries. A total of 121 non-CHD patients were included in the control group. Exclusion criteria are advanced cancer, severe liver or kidney dysfunction, congenital heart disease, severe aortic valve stenosis, and severe infection.

### Monocyte Isolation

Peripheral blood mononuclear cells (PBMCs) from CAD patients and control individuals were isolated by Ficoll-Paque PLUS density gradient centrifugation (GE Healthcare Bioscience). The PBMCs were cryopreserved for <3 months at −80°C in 90% heat-inactivated fetal bovine serum (FBS) (Sigma-Aldrich) and 10% DMSO (Sigma-Aldrich). Monocytes were isolated using the EasySep Human Monocytes Isolation Kit (Stemcell Technology) and cultured in RPMI-1640 (Lonza) containing 2 mM glutaMAX (Gibco), 20 μg/mL gentamicin (Gibco), and 2% normal human serum type AB (Invitrogen).

### Purification and Quantification of sEVs

Plasma and cell-line sEVs were purified by differential centrifugation as described previously. Briefly, culture medium was subjected to sequential centrifugation steps of 800 × g and 2,000 × g. The resulting supernatant was filtered using 0.2-μm filter and concentrated 20 times using a Vivaflow 200 R crossflow unit (Sartorius) with a 100,000 kDa cutoff filter. Then the plasma and culture medium were centrifuged at 16,500 g for 30 min. Ultracentrifugation was performed at 100,000 × g for 16 h at 4°C. The supernatant was removed, and phosphate-buffered saline was added to the pellet for an overnight washing step. The resultant sEVs pellet was resuspended in phosphate-buffered saline and harvested for downstream analyses.

sEVs samples were quantified *via* Brownian diffusion-size analysis using a ZetaView nanoparticle-tracking analysis (NTA) instrument (Particle Metrix). Sample aliquots were diluted 10^2^-10^6^-fold to achieve optimal concentration for analysis; 1.0 mL of diluted sample was used for each analysis. Light scattering of individual particles in solution was digitally recorded, particle trajectory and displacement were automatically analyzed using image analysis tracking software, and the particle-size distribution was determined from the observed brownian motion of individual particles according to the Stokes–Einstein relationship.

### mRNA Microarray and Computational Analyses

Total RNA was were amplified and transcribed into fluorescent cRNA utilizing a random priming method. The labeled cRNAs were hybridized onto the Human 4 × 180 K mRNA Array (Shanghai Biotechnology Corporation, Shanghai, China). After the arrays were scanned, the images were analyzed using the Agilent Feature Extraction software (version 11.0.1.1). Quantile normalization and subsequent data processing was performed using the limma package in R. mRNAs differentially expressed between the two types of samples were identified using volcano plot and fold change filtering. mRNAs with a fold-change ≥1.5 and an adjust-*P*-value (FDR) <0.05 in the microarray data were considered significantly differentially expressed. Hierarchical clustering was performed to test whether the two types of samples were distinguishable based on mRNA expression patterns.

### Quantitative Real-Time PCR (qRT-PCR)

Total RNAs were reverse-transcribed into cRNA with random primers using the Transcriptor First Strand cDNA Synthesis Kit (Roche, Penzberg, Germany) following the manufacturer's instructions. mRNA expression was measured with qRT-PCR using the FastStart Essential DNA Green Master (Roche, Penzberg, Germany) on the Roche LightCycler 480 (Roche, Penzberg, Germany). RNA expression was normalized to human GAPDH. All quantitative PCRs were run in triplicate. Primers are listed in Supplementary Table S1.

### Data Analysis

SPSS v25.0 was used for statistical analysis. Non-parametric (non-normally distributed) variables are expressed as quartiles and were analyzed with the Mann-Whitney U test. Parametric variables are expressed as mean ± standard deviation (X ±s). Measurement data with homogeneity of variance were analyzed with the independent-sample *t*-test. Count data were analyzed with the χ^2^ test. Spearman analysis was performed to analyze the correlation between two most differentially expressed mRNAs and clinical indicators, and binary logistic regression analysis was performed to analyze the independent correlation between each of the two mRNAs and CHD. The receiver operating characteristic (ROC) curve was used to analyze the value of sphingosine-1-phosphate receptor 5 (S1PR5) and carnosine synthase 1 (CARNS1) mRNA levels for diagnosing CHD. P <0.05 was considered statistically significant. We corrected *P*-values by the Benjamini–Hochberg method.

## Data Availability Statement

Publicly available datasets were analyzed in this study. This data can be found here: The obtained circRNA microarray datasets were deposited with the NCBI Gene Expression Omnibus (GEO) repository under accession number GSE166126.

## Ethics Statement

This study was approved by the Third People's Hospital of Chengdu Ethics, with registration number (2019) S−93. The study was conducted in accordance with the Declaration of Helsinki. The informed consent was obtained from each participant before the collection of blood samples. The patients/participants provided their written informed consent to participate in this study.

## Author Contributions

FX, TZ, and RZ conceived the project and designed the experiments. FX, RM, and RZ carried out the experiments. TZ wrote the manuscript and contributed to manuscript revision. KT and RM carried out the statistical analysis. YL provided assistance in collecting tissue samples. All authors provided suggestions during manuscript preparation and read the final version.

## Funding

This study was supported by Sichuan Science and Technology Department Project Fund (No. 2018JY0385).

## Conflict of Interest

The authors declare that the research was conducted in the absence of any commercial or financial relationships that could be construed as a potential conflict of interest.

## Publisher's Note

All claims expressed in this article are solely those of the authors and do not necessarily represent those of their affiliated organizations, or those of the publisher, the editors and the reviewers. Any product that may be evaluated in this article, or claim that may be made by its manufacturer, is not guaranteed or endorsed by the publisher.
