# Supplementary material for: Plasma Exosomal S1PR5 and CARNS1 as Potential Non-invasive Screening Biomarkers of Coronary Heart Disease
Source: Front Cardiovasc Med. 2022 Jun 28;9:845673. doi: 10.3389/fcvm.2022.845673 (PMC9273894; doi:10.3389/fcvm.2022.845673)
Supplement: Supplementary file 1 [file Table_1.docx]

Table S1 Primers used in the study

| Gene | Sequence | Product length  （bp） |
| --- | --- | --- |
| S1PR5 | 5’-GTGAGGTGGGAGCCATAGAA-3’ | 180 |
|  | 3’-TCTAGAATCCACGGGGTCTG-5’ |  |
| CARNS1 | 5’-CCAGTCCAGCCTACAGCTTC-3’ | 230 |
|  | 3’-TCAGGTTTTCCCAGGATTTG-5’ |  |
| C1orf162 | 5’-CGTGCTATGACCGGACTTTT-3’ | 208 |
|  | 3’-GGCTATCAGCAGCAGTGTCA-5’ |  |
| GDPD2 | 5’-CGCTGAGCAAAGATTCCTCT-3’ | 190 |
|  | 3’-CAGCATAATGAGGAGCAGCA-5’ |  |
| RASGRP4 | 5’-AATGCACCGGAAAAATAGGA-3’ | 179 |
|  | 3’-AGCTGAATCGAAGGACTGGA-5’ |  |
| HMGN4 | 5’-CCACGAGACTCGGACATCTT-3’ | 207 |
|  | 3’-CTCTGGTTTTGGAGGAGCTG-5’ |  |
| BCL3 | 5’-ACTACCCCGGAGCCTTACTG-3’ | 179 |
|  | 3’-AGCAATATGGAGAGGCGTGT-5’ |  |
| GHRHR | 5’-GTGGGTGAGCTGCAAACTG-3’ | 159 |
|  | 3’-TCCAGTTTCCTCACCAGGAT-5’ |  |
| GPN2 | 5’-GCAGCATCTTCTCCCAAATG-3’ | 200 |
|  | 3’-GAAGGCCAGCTTCCCATAAT-5’ |  |
| ZSCAN10 | 5’-CTGCCTGTGTAGTGGGGACT-3’ | 243 |
|  | 3’-CTTTGAACTTGGGGGAAGC-5’ |  |
| GAPDH | 5’-GAAAGCCTGCCGGTGACTAA-3’ | 150 |
|  | 3’-GCCCAATACGACCAAATCAGAG-5’ |  |
